# Which training load indicators are greater correlated with maturation and wellness variables in elite U14 soccer players?

**DOI:** 10.1186/s12887-024-04744-9

**Published:** 2024-04-30

**Authors:** Hadi Nobari, Özgür Eken, Utkarsh Singh, Armin Gorouhi, José Carlos Ponce Bordón, Pablo Prieto-González, Ahmet Kurtoğlu, Tomás García Calvo

**Affiliations:** 1 Faculty of Sport Sciences, University of Extremadura, Cáceres, 10003 Spain; 2 Department of Exercise Physiology, Faculty of Educational Sciences and Psychology, University of Mohaghegh Ardabili, Ardabil, 56199-11367 Iran; 3 Physical Education and Sports Teaching, Faculty of Sport Science, Inonu University, Battalgazi, Malatya, Türkiye; 4 Sport and Exercise Science, College of Healthcare Sciences, James Cook University, Townsville, Australia; 5 University of A Coruña, A Coruña, 15001 Spain; 6 Health and Physical Education Department, Prince Sultan University, Riyadh, 11586 Saudi Arabia; 7 Department of Coaching Education, Faculty of Sport Science, Bandirma Onyedi Eylul University, Balikesir, 10200 Türkiye; 8 Sports Dynamix Private Limited, Chennai, India; 9 Physical Activity and Health Promotion, Department of Biomedicine, University of Tor Vergata, Rome, Italy

**Keywords:** Football, Maturation, Monotony, Psychological states, RPE, Youth sports

## Abstract

**Background:**

Monitoring of training load is done to improve physical performance and minimize the incidence of injuries. The study examined the correlation between accumulated training load parameters based on periods with maturity (i.e., maturity offset and peak height velocity -PHV- and wellness variables -e.g., stress and sleep quality-). The second aim was to analyze the multi-linear regression between the above indicators.

**Methods:**

Twenty elite young U14 soccer players (M = 13.26 ± 0.52 years, 95% CI [13.02, 13.51]) were evaluated over 26 weeks (early, mid, and end-season) to obtain stress, sleep quality, and measures of workload in the season (accumulated acute workload [AW], accumulated chronic workload [CW], accumulated acute: chronic workload ratio [ACWLR], accumulated training monotony [TM], accumulated training strain [TS]).

**Results:**

The analysis revealed a moderate, statistically significant negative correlation between sleep quality and training monotony (*r* = -0.461, *p* < 0.05). No significant correlations were observed between other variables (*p* > 0.05). In the multi-linear regression analysis, maturity, PHV, sleep, and stress collectively accounted for variances of 17% in AW, 17.1% in CW, 11% in ACWLR, 21.3% in TM, and 22.6% in TS. However, individual regression coefficients for these predictors were not statistically significant (*p* > 0.05), indicating limited predictive power.

**Conclusion:**

The study highlights the impact of sleep quality on training monotony, underscoring the importance of managing training load to mitigate the risks of overtraining. The non-significant regression coefficients suggest the complexity of predicting training outcomes based on the assessed variables. These insights emphasize the need for a holistic approach in training load management and athlete wellness monitoring.

## Introduction

In team sports training, training load (TL) has been defined as the input variable manipulated to induce the desired training response [1]. Training load can be classified into two categories, namely, external and internal load [2]. The external training load (EL) refers to the work done by the athletes in terms of running distance, the number of sprints performed, and the number of acceleration/decelerations runs, for example, performed during training or matches. Global positioning systems (GPS) or inertial sensors are commonly used for quantifying and monitoring EL during practice sessions and competitive matches. The internal training load corresponds to the indicators reflecting the psychophysiological response that the body initiates to meet up the requirement imposed by the EL. Several measures, such as rating of perceived exertion (RPE), session RPE, oxygen uptake, heart rate response, etc., [2] can be used for measuring internal load. The main aim of TL is to improve physical performance [3], minimize the incidence of injuries [4] and reduce the risk of nonfunctional overreaching [5] in athletes. A soccer season involves several fluctuations in training measures, and it is essential to monitor them to design and implement an optimal recovery strategy [6]. Also, coaches and practitioners can use this information to modify or implement their sessions and ensure that the optimal training dosage is administered to the players for competitive matches without exposing them to an increased risk of injury.

The increasing participation and specialization of youth in a particular sport have made TL monitoring essential for youth soccer players [7]. In particular, quantifying TL is even more important in youth athletes since they have a higher risk of sustaining injuries during various stages of growth and maturation [7]. This is primarily attributed to the high TL undergone by young soccer players, which coincides with their rapid changes in growth. Further, these injuries have risen following increased or reduced training exposure [8–10]. Studies in elite youth football have analyzed the dependency amongst training load variations and maturation variables [11, 12]. In this vein, Nobari et al. [11] reported the effects of accumulated training load and maturation status in the differences observed across the season. Therefore, understanding the association between maturity and TL will provide valuable information for practitioners that might help manage training programs considering the maturity level of young soccer players. For instance, subjective well-being questionnaires measure athletes’ training readiness [5, 14]. These questionnaires generally provide information regarding soreness, mood, fatigue, strain, and stress levels [13]. Previous studies have explored the relationship between training load and perceived wellness rating. A recent study by Nobari et al. [14] reported small to significant correlations between TL and well-being measures in Under-16 soccer players. Another study on Under-16 soccer players revealed that wellness indicators (fatigue, DOMS, or stress) were primarily correlated with weekly acute TL [15]. However, there is still a paucity of literature regarding the association between well-being measures and TL in youth soccer players. Therefore, further research exploring the association between well-being measures and TL will help practitioners learn about weekly session distribution and workload responses during practice and competitive matches.

Along with TL, poor sleep quality has been associated with an increased risk of injury and overtraining syndrome [16, 17]. A few studies have examined the relationship between sleep quality, sleep duration, and training load. For example, Watson et al. [18] reported that increased training load was linked with decreased sleep duration and quality in female soccer players. Likewise, Pitchford et al. [19] reported that sleeping quality and duration are affected by changes in training load in Australian rules football players. Moreover, sleep quality monitoring was shown to be sensitive to daily fluctuations in training loads in elite soccer players [20]. Therefore, the above findings related to sleep and training loads are essential to optimize sports performance, health, and well-being.

However, the current findings highlight that there still needs more evidence regarding the relationship between TL and stress, sleep, and maturity in youth soccer players. Further research aimed at understanding the associations between TL, maturity, and well-being measures may provide practitioners and coaches with further evidence regarding the management of load during training sessions and its influence on sleep quality and stress, which can impair performance [4]. Therefore, this study aimed to examine the association between training load parameters based on periods (early, mid, and end-season) with maturity, stress, and sleep quality. This study posits that there exists a significant association between training load parameters, categorized by different periods within the soccer season (early, mid, and end-season), and the well-being of youth soccer players, encompassing stress levels and sleep quality. It is anticipated that fluctuations in training load throughout the season will exhibit correlations with variations in perceived stress and sleep quality among the players. This hypothesis is grounded in the notion that understanding the intricate relationship between training load and psychological and physiological well-being is crucial for practitioners and coaches in tailoring effective training programs that optimize both performance and the overall health of youth soccer players.

## Materials and methods

### Participants

A sample size estimation was conducted through a statistical power analysis. The effect size (ES) in our study was established using G Power software (Version 3.1), wherein we computed the coefficient of determination (R^2^ > 0.5 based on values reported in prior studies [21, 22]. With a significance level of alpha = 0.05 and a power of 0.80, the anticipated sample size required for the most basic correlational analysis was determined to be 20. Therefore, twenty elite young football players comprised this study’s sample (Mean ± Standard deviation; chronological age: 13.26 ± 0.52 years; height: 165.80 ± 11.67 cm; body mass:50.70 ± 7.56 kg; peak height velocity: 13.26 ± 0.20 years; maturity offset: -0.01 ± 0.56 years; VO_2max_, 48.22 ± 2.29 ml.kg^− 1^.min^− 1^).

The age category of participants was U14, and according to the relevant federation’s program, they competed first in the regional league and then the national league. Four players were central defenders, four were central midfielders, four were wide defenders, five were wide midfielders, and three were attackers. Inclusion criteria were (1) at least three years of soccer experience. Longitudinal engagement in soccer provides players with a more extensive and varied exposure to training loads, match conditions, and overall soccer-related activities. This extended experience contributes to the development of specific physiological adaptations, technical skills, and tactical understanding, making these players more representative of the elite youth soccer population [23, 24] ; (2) active and regular participation in all the activities included in the study; (3) do not receive any supplements that could affect their growth or maturation. Testosterone boosters [25], synthetic anabolic steroids [26], and growth hormone supplements [27], marketed for performance enhancement, pose risks to adolescent growth and maturation by disrupting hormonal balance. High caffeine doses in pre-workout supplements can impact sleep patterns, affecting development [27]. Ephedrine-containing supplements, used for weight loss, may interfere with cardiovascular and central nervous system functions in young athletes [28]; and (4) do not practice additional physical activities aside from those included in this study. The exclusion criteria were: (1) do not participate in 80% of the competitions (official and non-official) and training sessions during the season. The criterion mandating at least 80% participation in competitions and training sessions is essential for reliable data and meaningful interpretations. It aligns with sports science principles, ensuring athletes’ adaptation, skill development, and injury prevention. Inconsistent participation introduces variability, hindering the study’s validity and the athletes’ representation [29–31]; (2) do not attend any of the medical or physical examinations conducted. The data of 4 athletes who did not meet the inclusion and exclusion criteria of our study were not included in the analyses. Each week, if any player competed for a short amount of time throughout the match. Then, we presented an exhibition game or a small-sided game.

This research was authorized by the University of Mohaghegh Ardabili Ethical Committee and was conducted according to the Helsinki declaration [32]. The ethical reference number is 10.07.2021. All participants were informed of the risks and benefits of this study and had the option to withdraw at any time. The parents/young participants signed a consent-informed agreement to participate in the study.

### Study design

**Fig. 1:**
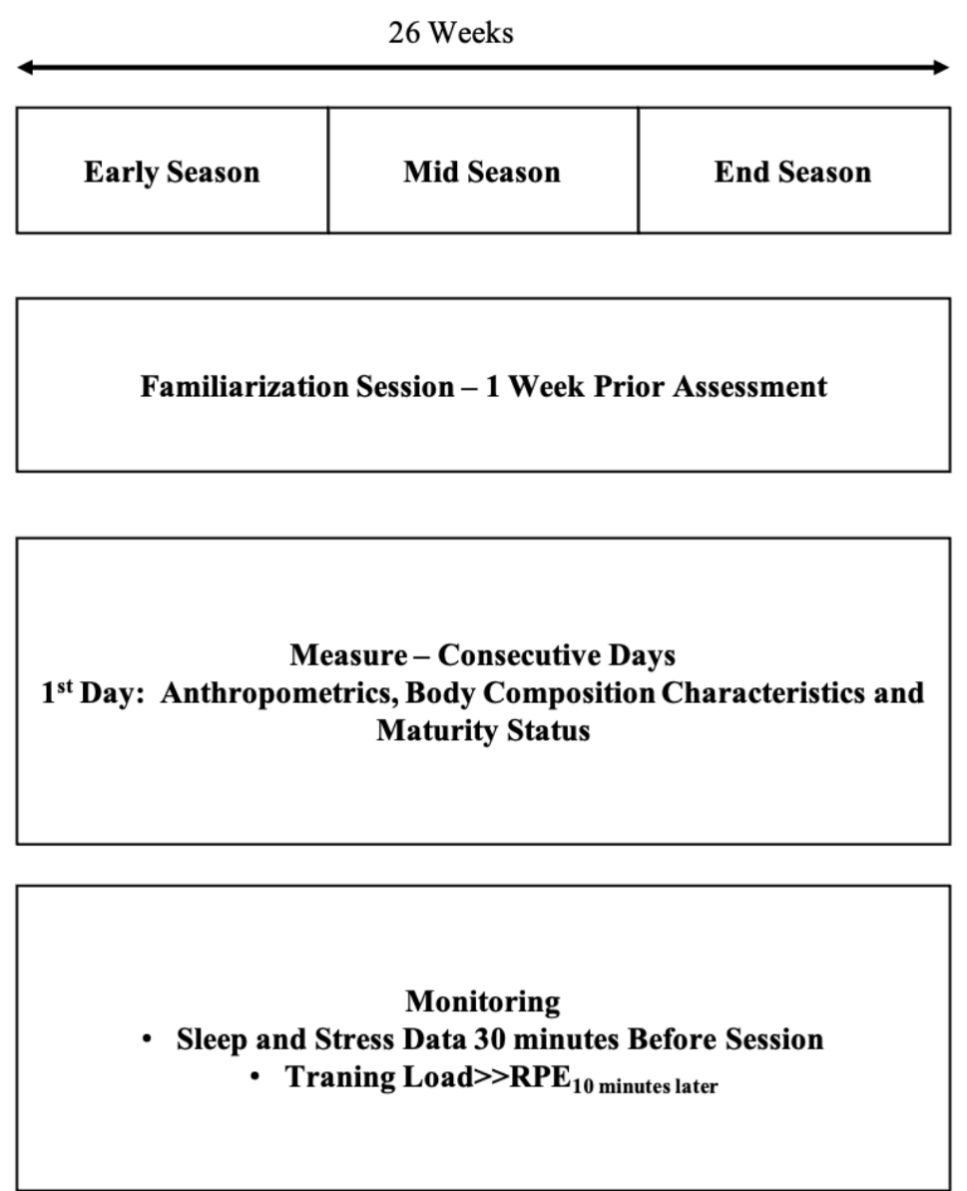
Schematic diagram of research design

This research was conducted as a prospective study with an observational cohort design, which was performed on a cross-sectional basis, yielding practical results. Researchers have checked players over the whole season, and assessments were performed upon completion of the competitive season. The present study was conducted over the 26-week. We divided the season into three equal periods (early, mid, and end-season). Players were measured on consecutive days during each test. On the first day of testing, anthropometrics, body composition characteristics, and maturity status were used to calculate each player’s age at PHV. Approximately thirty minutes before sessions, players provided the stress and sleep quality status based on Hooper index questioners [33] with the same procedures of the RPE at the end of the training session. Daily average data was used for each category. A familiarization session was organized one week before the evaluation. For this cohort study, all participants reported the training load 30 min after each training session, and each “training load” was then calculated alongside the training time to determine the accumulated effort for every period (Fig. 1).

### Anthropometric and maturity

All anthropometric and body composition measurements were performed during the morning. Subject’s height and sitting height was measured by a skilled person using a stadiometer (Seca model 213, Germany) with a precision of 5 mm and weight was measured and recorded with a digital scale (Seca model 813, UK) with a precision of 0.1 per kg. Based on the information collected above and using the Mirwald formula, the maturity o set and age at PHV was determined [34]. The formula used is as follows: maturity offset = − 9.236 + 0.0002708 (leg length × sitting height) − 0.001663 (age × leg length) + 0.007216 (age × sitting height) + 0.02292 (weight by height ratio), where *R* = 0.94, R2 = 0.891, and SEE = 0.592) and for leg length = standing height (cm) - sitting height (cm).

#### Monitoring workloads training

Half an hour after training, each player was asked, “How intense was the training?” for each session on a Borg Category-Ratio-10 scale. On this scale, one a short training session, and then a very high-intensity training session [24]. The TL was then calculated considering the s-RPE and training time for each training session. These data were used to obtain information and analyze the weekly workload parameters (accumulated acute workload [AW], accumulated chronic workload [CW], accumulated acute: chronic workload ratio [ACWLR], accumulated training monotony [TM], accumulated training strain [TS]) [35, 36]. Thus, the following calculations were made: [37–39].

*ACWLR = acute workload(most recent week)∕chronic workload(last 4 weeks)*

*TM = mean training load during the seven days of the week∕standard deviation of training load during the seven days of the week*

*TS = sum of the training loads for all weekly sessions × TM*

### Aerobic power test

The study utilized the Intermittent Fitness Test 30 − 15 (30-15IFT) to assess VO2max and subjects’ readiness [40, 41]. The test involved 30s shuttle runs with 15s recovery periods, starting at 8 km/h with 0.5 km/h increments. After a 10-minute warm-up, subjects ran back and forth between lines 40 m apart, adjusting pace to prerecorded beeps. The test continued until subjects couldn’t proceed or failed three consecutive attempts to reach lines. VO2max (ml kg–1 min–1) was determined using the formula: 28.3 – (2.15 × 1) – (0.741 × 16 years) – (0.0357 × weight) + (0.0586 × 16 years × VIFT) + (1.03 × VIFT). VIFT represented the final running speed. Test–retest reliability was calculated with an ICC of 0.86 [41].

### Monitor sleep and stress

Hooper index is a questionnaire that includes fatigue, stress, DOMS, and quality of sleep (scale of 1–7, in which 1 is very low and 7 is very high). We only considered sleep and stress in the present study. This questionnaire was applied 30 min before each session. The players were familiarized with the scale before the study. For each variable, the sum of a week was used to obtain the data mentioned above. Data were collected separately to prevent the players from hearing their teammates’ scores. An excel file was used to create the daily data register.

#### Statistical analysis

Statistical analyses were performed using GraphPad Prism 8.0.1 (GraphPad Software Inc, San Diego, California, USA). The significance level was set at *p* < 0.05. Data are presented as mean and SD. Shapiro–Wilk was applied to check the normality of the data, and Levene’s test the homoscedasticity. Pearson correlation analysis was performed between training load parameters (AW, CW, ACWLR, TS, and TM) periods using maturity, PHV, sleep, and stress factors. Repeated Measures Correlation analysis of the relationship between repeated measures of training load parameters (AW, CW, ACWLR, TS and TM) using maturity, PHV, sleep and stress factors was performed with R 4.2.5 (Auckland University, New Zealand) [42]. The effect size of the correlations was determined by considering the following thresholds [43, 44]: <0.1 = trivial; 0.1–0.3 = small; > 0.3–0.5 = moderate; > 0.5–0.7 = large; > 0.7–0.9 = very large; and > 0.9 = nearly perfect. Then, multiple linear regression analysis was performed between training load parameters (AW, CW, ACWLR, TS, and TM), with variations in maturity, PHV, sleep, and stress. The intended regression type was least-squares.

## Results

The descriptive characteristics of the players are shown in Table 1. In the whole season, the accumulated AW was 1284.56 ± 68.13 Arbitrary unit (A.U.), accumulated CW was 1283.29 ± 73.75 (A.U.), accumulated ACWLR was 1.01 ± 0.10 (A.U.), accumulated TM was 4.43 ± 0.64 (A.U.), accumulated TS was 5693.55 ± 861.21 (A.U.).

**Table 1:** Descriptive characteristics of soccer players (M ± SD)

Variables	Mean ± SD	95% CI Lower	95% CI Upper
Height (cm)	165.80 ± 11.67	160.34	171.26
Body mass (kg)	50.70 ± 7.56	47.16	54.24
Sitting height (cm)	87.85 ± 6.37	84.87	90.83
Age at PHV (years)	13.26 ± 0.52	13.02	13.51
Maturity Offset (years)	-0.01 ± 0.55	-0.27	0.25
Age (years)	13.25 ± 0.20	13.16	13.34
VO2max (mL.kg-1.min-1)	44.23 ± 2.80	42.91	45.54
Body Fat (%)	70.49 ± 4.99	68.15	72.82
AW (A.U.)	1284.56 ± 68.13	1252.68	1316.45
CW (A.U.)	1283.30 ± 73.75	1248.78	1317.81
ACWLR (A.U.)	1.02 ± 0.11	0.97	1.07
TM (A.U.)	4.43 ± 0.65	4.13	4.73
TS (A.U.)	5693.55 ± 861.21	5290.49	6096.61

*Note* PHV = peak height velocity; VO_2max_ = maximal oxygen consumption; AW = accumulated acute workload in the season; CW = accumulated chronic workload in the season; ACWLR = accumulated acute: chronic workload ration in the season; TM = accumulated training monotony in the season; TS = accumulated training strain in the season, and A.U. =Arbitrary unit

**Table 2:** Analysis of the correlation between training load parameters (AW, CW, ACWLR, TS, and TM) based on periods (early, mid, and end season) and maturity (maturity offset and PHV), sleep quality, and stress variable

Variables	PHV	Maturity	AW1	AW2	AW3	CW1	CW2	CW3	ACWLR1	ACWLR2	ACWLR3	TM1	TM2	TM3	TS1	TS2	TS3	Stress	Sleep
**PHV**	1																		
Maturity	− 0.935**	1																	
AW1	0.247	− 0.419	1																
AW2	0.095	− 0.33	0.797**	1															
AW3	0.285	− 0.197	0.724**	0.756**	1														
CW1	0.193	− 0.05	0.962**	0.792**	− 0.695*	1													
CW2	0.053	− 0.3	0.802**	0.99**	0.754**	0.796**	1												
CW3	0.266	− 0.066	− 0.757**	0.831**	0.986**	0.727**	0.822**	1											
ACWLR1	− 0.318	0.226	− 0.401	− 0.105	− 0.316	− 0.502*	− 0.140	− 0.256	1										
ACWLR2	0.424	− 0.406	0.305	0.224	0.338	0.276	0.276	0.317	− 0.204	1									
ACWLR3	0.154	− 0.067	− 0.038	0.084	− 0.268	− 0.002	0.055	− 0.217	− 0.055	0.214	1								
TM1	− 0.126	− 0.051	− 0.145	0.061	− 0.333	− 0.174	0.037	− 0.236	0.677*	− 0.022	0.352	1							
TM2	0.171	− 0.327	0.278	0.537*	0.184	0.305	0.555	0.232	0.051	0.329	0.345	0.359	1						
TM3	0.227	− 0.11	− 0.098	− 0.140	0.162	− 0.079	− 0.153	− 0.082	− 0.008	0.563*	0.569*	0.152	0.138	1					
TS1	0.100	− 0.232	0.311	0.434	0.01	0.285	0.410	0.12	0.340	0.155	0.444	0.868**	0.528*	0.133	1				
TS2	0.143	− 0.396	0.471	0.745**	0.356	0.5	.76**	0.437	− 0.016	0.328	0.304	0.316	0.958**	0.055	0.573*	1			
TS3	0.273	− 0.142	0.065	− 0.002	0.153	0.078	− 0.014	0.114	− 0.106	0.609*	0.483	0.034	0.11	0.969**	0.087	0.075	1		
Stress	− 0.155	0.023	0.033	0.087	0.250	0.032	0.053	0.266	− 0.318	0.424	0.154	− 0.126	0.171	0.227	0.1	0.143	0.273	1	
Sleep	− .999**	0.151	0.249	0.015	0.287	0.196	0.098	0.219	− 0.197	0.339	0.239	− 0.127	0.095	0.483	− 0.067	0.1	0.535*	**− .1**56	1

*Note* Significant differences (*p* ≤ 0.05) are highlighted in bold

PHV = Peak height velocity; AWL = acute workload; CWL = chronic workload; ACWLR = acute: chronic workload ratio; TM = training monotony; and TS = training strain; and 1, 2, and 3 = mean differences between assessments (EaS to MiS, and MiS to EnS, and EaS to EnS), respectively

**Fig. 2:**
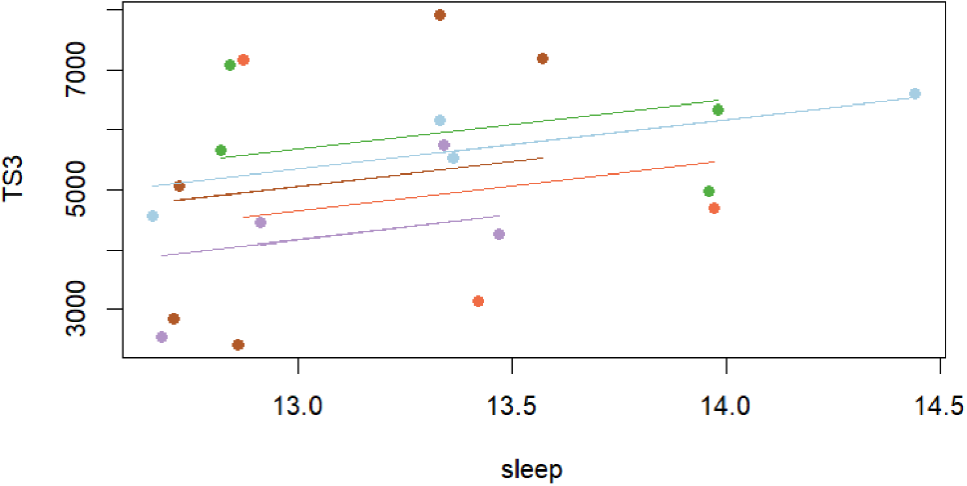
Repeated measures correlation test results between sleep and TS3: TS: Training Strain

Table 2 shows the repeated measures correlation analysis between TL parameters (AW, CW, ACWLR, TS and TM) and maturity (maturity offset and PHV), stress and sleep variables based on periods (early, mid and end of season). The following results were obtained in the correlations between training load parameters and maturity stress and sleep variables according to the periods: PHV was correlated with maturity (*r* = − 0.935, nearly perfect) and sleep (*r* = 0.999, nearly perfect), sleep and TS3 (*r* = 0.533, moderate) (Fig. 2).

**Table 3:** Analysis of correlation between the total amount of training load parameters based on maturation and wellness variables

Variables	Sleep	Maturity offset	PHV	Stress	AW-Total	CW-Total	ACWLR-Total	TM-Total	TS-Total
Sleep	1								
Maturity offset	-0,935**	1							
PHV	0,020	-0,119	1						
Stress	-0,262	0,165	0,235	1					
AW-Total	0,200	-0,231	0,221	-0,079	1				
CW-Total	0,118	-0,189	0,385	0,033	0,921**	1			
ACWL-Total	-0,206	0,278	-0,161	-0,018	-0,625**	-0,591**	1		
TM-Total	0,424	-0,412	0,060	0,227	-0,161	-0,082	0,189	1	
TS-Total	0,439	-0,465*	0,189	0,260	0,230	0,348	-0,037	0,892**	1

*Note* Significant differences (*p* ≤ 0.05) are highlighted in bold

PHV = Peak height velocity; AWL-Total = total amount of acute workload; CWL-Total = total amount of chronic workload; ACWLR-Total = total amount of acute: chronic workload ration; TM-Total = total amount of training monotony; and TS-Total = total amount of training strain

Table 3 shows the analysis of the correlation between the total amount of training loads parameters (AW-Total, CW- Total, ACWLR- Total, TS-Total, and TM- Total) based on maturation (i.e., maturity offset and PHV), wellness variables (i.e., sleep quality and stress). The results showed maturity offset to sleep (*r* = -0.935 nearly perfect, CI 95% {-0.53 to 0.34} is nearly perfectly related. Also, maturity offset to TS-Total (*r* = -0.46 moderate, CI 95% {-0.75 to − 0.02}; *p* = 0.03) is moderately related.

**Table 4:** Multiple linear regression analysis: Percentage of variation between training load parameters with maturity and sleep variables

Variable	Beta	Estimate	|t|	p Value	95% CI for Estimated	
**AW**	β0	827,6	0,5684	0,5782	-2276 to 3931	***R***^2^ = 0,1705 Adjusted ***R***^2^ =-0.05 *p* = 0.56 **AIC** = 182.6
Sleep	β1	-15,89	0,1710	0,8665	-214,0 to 182,2	
Maturity offset (years)	β2	-39,07	0,4503	0,6589	-224,0 to 145,9	
PHV (years)	β3	46,33	1,208	0,2456	-35,40 to 128,1	
Stress	β4	38,86	0,3177	0,7551	-221,8 to 299,5	
**CW**	β0	817,2	0,5188	0,6115	-2540 to 4175	***R***^2^ = 0.1715 Adjusted ***R***^2^ =-0.04 *p* = 0.55 **AIC** = 185.7
Sleep	β1	-25,61	0,2547	0,8024	-239,9 to 188,7	
Maturity offset (years)	β2	-37,41	0,3986	0,6958	-237,5 to 162,7	
PHV (years)	β3	60,34	1,455	0,1664	-28,08 to 148,8	
Stress	β4	4,238	0,03203	0,9749	-277,8 to 286,2	
**ACWLR**	β0	0,3447	0,1434	0,8879	-4,779 to 5,468	***R***^2^ = 0.1100 Adjusted ***R***^2^ =-0.12 *p* = 0.76 **AIC** = − 73.70
Sleep	β1	0,07117	0,4639	0,6494	-0,2559 to 0,3982	
Maturity offset (years)	β2	0,1177	0,8216	0,4241	-0,1876 to 0,4230	
PHV (years)	β3	-0,02000	0,3160	0,7564	-0,1549 to 0,1149	
Stress	β4	-0,003823	0,01894	0,9851	-0,4342 to 0,4265	
**TM**	β0	-4,845	0,3600	0,7238	-33,53 to 23,84	***R***^2^ = 0.2133 Adjusted ***R***^2^ = 0.00 *p* = 0.43 **AIC** = -4.808
Sleep	β1	0,5789	0,6741	0,5105	-1,252 to 2,410	
Maturity offset (years)	β2	0,1105	0,1378	0,8923	-1,599 to 1,820	
PHV (years)	β3	-0,06782	0,1914	0,8508	-0,8231 to 0,6875	
Stress	β4	1,818	1,608	0,1286	-0,5912 to 4,227	
**TS**	β0	-6826	0,4034	0,6924	-42,893 to 29,241	***R***^2^ = 0.2989 Adjusted ***R***^2^ = 0.11 *p* = 0.22 **AIC** = 280.7
Sleep	β1	575,6	0,5330	0,6019	-1726 to 2878	
Maturity offset (years)	β2	-142,8	0,1416	0,8893	-2292 to 2006	
PHV (years)	β3	98,28	0,2206	0,8284	-851,5 to 1048	
Stress	β4	2608	1,835	0,0864	-421,6 to 5637	

**Fig. 3:**
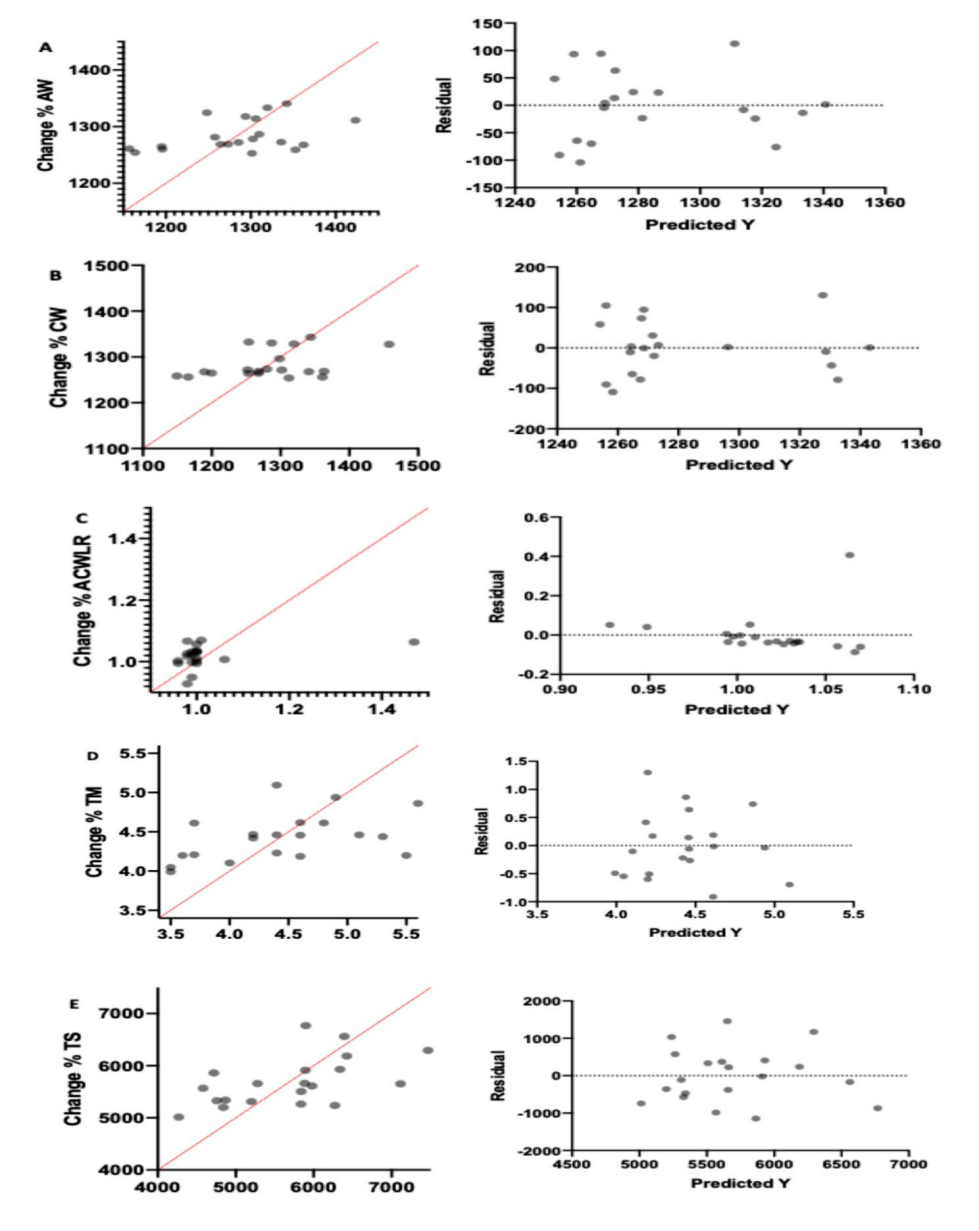
Multiple linear regression analysis was calculated to predict the percentage of change between training load parameters with maturity and sleep quality. (a); AW (A.U.) = acute workload; (b); CW (A.U.) = chronic workload; (c); ACWLR (A.U.) = acute: chronic workload ration; (d); TM (A.U.) = training monotony; (e); TS (A.U.) = training strain; A.U. =Arbitrary unit

Multiple linear regression was used to find independent predictors of training load parameters (AW, CW, ACWLR, TS, and TM) periods using maturity, PHV, sleep, and stress factors. However, their coefficients were not determined to be statistically significant (*p* > 0.05) (Table 4; Fig. 3).

## Discussion

This study aimed to examine the relationship between accumulated TL parameters, maturation, and wellness markers in youth soccer players during different times of an entire competitive season. As for the association between parameters (AW, CW, ACWLR, TS, and TM) based on periods (early, mid, and end season) and maturity (maturity offset and PHV), sleep quality, and stress variable, only TM at early season had significant correlation with sleep (*r*= -0.461 with moderate effect). Previous studies also conducted with young and adult soccer players, both professional and amateur, have demonstrated that durations of increased workloads increase the amount of disruption related to sleep outcomes (e.g., decreased sleep duration and quality) [45]. Figueiredo et al. [46] and Costa et al. [47] found that during a competitive two-week period for high-level female soccer players and during an international training camp for youth male soccer players, the workloads at the lowest and highest levels affected sleep durations. It was also considered when training and matches were performed in the evening, close to sleeping time. The correlation between sleep quality and TM in this research was *p* = 0.041. In contrast, in a similar study by Knufinke and colleagues [48] about sleeping quality and quantity and their relationship with the training load of elite athletes, athletes’ sleeping parameters were recorded at low, medium and high training loads. With medium and high training loads, it was verified that sleep time (*p* = 0.75) and sleep efficiency (*p* = 0.15) had no significant correlations with training load.

According to other similar studies, these findings suggest that male youth athletes who train specifically at PHV-related ages (during or after), may experience an improved training response because their anabolic hormone levels are higher. This reaction subsequently enhances strength and sprinting performance during and after PHV and provides a plausible defense for manipulating training volume concerning maturity [49]. Based on the acquired results, great correlations were found between TM and ACWR ( *r* = 0.669 ) in the first season. In a similar study by Nobari and colleagues [50], which examined the accumulated- training load parameters of young soccer players, no meaningful relationship between these factors was observed (*p* = -0.29).

When we analyzed the data for the entire season, we observed a similar result in the TS and maturity offset with a moderate correlation (*r* = -0.46, *p* = 0.03). In contrast, in a similar article conducted by Nobari and colleagues [51] it was found that the training load and maturity offset and other parameters such as muscle soreness and fatigue, PHV and TS had strong correlations (*r* = 0.506, *p* = 0.022). In the same study, maturity and TS were strongly correlated (*r* = 0.504, *p* = 0.023) in the second half of the season. However, they did not find correlations between other variables.

To our knowledge, no studies have examined the relationship between accumulated TL parameters, maturity, and wellness measures during different periods of an entire competitive season under 14 soccer players. In this study, we have investigated the relationship between well-being parameters and TL with different intensities in various micro-cycles during the competitive season. However, in some of these elements, no significant correlations were observed. In this vein, some limitations of the present research, such as the number of participants, may have affected the result. Even so, the results of this study may be helpful for future studies wherein researchers examine the effect of TL and its variations on wellness parameters.

## Conclusion

To conclude, the training monotony in the early season was significantly correlated with sleep. This finding can guide head coaches and strength and conditioning coaches in planning the training sessions, particularly when deciding whether to apply monotonic or non-monotonic (wave) increases and decreases in the training load. For that reason, overload states and injuries can be avoided.

Practical applications for practitioners from this study include emphasizing the importance of monitoring sleep quality in young soccer players as a key factor in managing training load. Given the significant negative correlation between sleep quality and training monotony, coaches and trainers should prioritize regular assessment of athletes’ sleep patterns and quality. This could involve implementing strategies such as sleep hygiene education and adjusting training schedules to enhance rest periods. Additionally, the findings suggest that relying solely on traditional training load metrics may not adequately predict wellness outcomes. Therefore, incorporating a holistic approach that includes both physical and wellness variables, such as stress levels and maturity indicators, can provide a more comprehensive understanding of each athlete’s needs. This approach can help in tailoring training programs that are both effective in improving performance and mindful of the athletes’ overall health and well-being.

## Data Availability

The data presented in this study are available on request from the corresponding author.

## Abbreviations

AW: Accumulated Acute Workload
CW: Accumulated Chronic Workload
ACWLR: Accumulated Acute: Chronic Workload Ratio
TM: Accumulated Training Monotony
TS: Accumulated Training Strain
